# Effect of β-sitosterol against methyl nitrosourea-induced mammary gland carcinoma in albino rats

**DOI:** 10.1186/s12906-016-1243-5

**Published:** 2016-07-29

**Authors:** Chetan Manral, Subhadeep Roy, Manjari Singh, Swetlana Gautam, Rajnish K. Yadav, Jitendra K Rawat, Uma Devi, Md Nazam Ansari, Abdulaziz S. Saeedan, Gaurav Kaithwas

**Affiliations:** 1 Department of Pharmaceutical Sciences, School of Biosciences and Biotechnology, Babasaheb Bhimrao Ambedkar University (A Central University), Vidya vihar, Raibareli road, Lucknow, 226025 (U.P.) India; 2 Department of Pharmaceutical Sciences, FHMSIASM SHIATS-Deemed University (Formerly Allahabad Agriculture Institute), Naini, Allahabad, 211007 (U.P.) India; 3 Department of Pharmacology, College of Pharmacy, Prince Sattam Bin Abdulaziz University, Al-Kharj, KSA

**Keywords:** β-sitosterol, Mammary gland, P-glycoprotein, Oxidative stress, Inflammation, FAME

## Abstract

**Background:**

The present study was in quested to study the effects of β-sitosterol on methyl nitrosourea (MNU) induced mammary gland carcinoma in albino wistar rats.

**Methods:**

Animals were randomized and divided into four groups of eight animals each. Group I (sham control 1 % CMC in normal saline p.o.); Group II (toxic control, MNU 47 mg/kg, i.v); Group III (MNU 47 mg/kg, i.v + β-sitosterol, 10 mg/kg, p.o); Group IV (MNU 47 mg/kg, i.v + β-sitosterol, 20 mg/kg, p.o). Toxicity was induced by single i.v. injection of MNU followed by β-sitosterol supplementation therapy for 115 days at the dose mentioned above.

**Results:**

Treatment with β-sitosterol evidenced decrease in the alveolar bud and lobule score in the whole mount of the mammary gland. β-sitosterol exhibited diminishing effect on oxidative stress through synchronizing lipid and enzymatic antioxidant defense. A significant decrease in the saturated and unsaturated fatty acid was evident with the MNU treatment and β-sitosterol demonstrated a marked effect on it. Pgp 9.5 expression was dose dependently upregulated by β-sitosterol treatment in comparison to MNU treatment. On the contrary, downregulated NF-kB expression was perceived, when β-sitosterol was concomitantly administered with MNU.

**Conclusion:**

β-sitosterol afforded significant protection against the deleterious effects of MNU.

## Background

Cancer is a one of the most life-threatening disease worldwide. Mammary gland cancer constitutes about 10 % of the total cancer population [1]. However alteration in cell membrane integrity, dysregulated apoptotic pathway along with other inflammatory changes are reported to play a crucial role [2, 3].

Recent findings have demonstrated that the dietary factors including fat and other phytochemicals may offer protection against mammary gland carcinoma, infact, controlled dietary preclinical studies are suggestive that the phytosterols may offer protection from a wide array of cancers including mammary gland carcinoma [4–7]. The phytosterols are the cholesterol-like compounds exclusively synthesized by plants, and it is hypothesized that phytosterols can induce apoptosis/programmed cell death in highly proliferating tumor cells [8–10]. Henceforth, β-sitosterol has been evaluated against a variety of cancers using preclinical experimental models [11, 12]. The studies have suggested potential efficacy of β-sitosterol particularly towards colon cancer, prostate cancer and leukemia [13–16]. On the same line, few preliminary studies have also demonstrated that β-sitosterol can induce apoptosis by activating fatty acid synthase (FAS) signaling in MCF-7 cells [17].

Additionally, Pgp-9.5 (ubiquitin COOH-terminal esterase L1 or UCHL-1) is an ubiquitin COOH terminal hydrolase that is widely expressed in different type of cancer cells [18]. Recent study demonstrated that Pgp-9.5 is highly overexpressed at many non-neuronal tumors, including breast, colorectal and pancreatic tumors [19].

Considering the same, the present study was undertaken to study the effect of β-sitosterol on Pgp expression and cellular lipids in methyl nitrosourea (MNU) induced mammary gland carcinoma in female albino rats. The study also extends its horizon towards elucidating the biochemical and inflammatory paradigms as markers for efficacy of β-sitosterol against MNU induced mammary gland carcinoma.

## Methods

### Drugs and chemicals

MNU was purchased from Sigma-Aldrich Co. St. Louise Mo 63103 USA. β-sitosterol was purchased from Cayman Chemical Company, Michigan, USA. All other chemicals were of analytical grade and obtained from Himedia Laboratories, Mumbai, India, else otherwise stated in the text.

### Experimental protocol

Albino wistar female rats of 100–120 g body weight were used for the study. The rats were procured from the central animal house facility. The animals were housed in propylene cages under controlled conditions (23 °C, 12 h light/ dark cycle) with free access to commercial pellet diet and water. Animals were acclimatized for two weeks before the commencement of the experiment. Animals were randomized and divided into 4 groups of 8 animals each. Group I (sham control 1 % CMC in normal saline p.o.); Group II (toxic control, MNU 47 mg/kg, i.v); Group III (MNU 47 mg/kg, i.v + β-sitosterol, 10 mg/kg, p.o); Group IV (MNU 47 mg/kg, i.v + β-sitosterol, 20 mg/kg, p.o). Toxicity was induced by single i.v. injection of MNU followed by β-sitosterol supplementation therapy for 115 days at the dose mentioned above. The blood samples were collected under chloroform anesthesia through retro-orbital plexus in centrifugation tubes. The blood samples were incubated at 37 °C for 1 h and centrifuged at 10,000 rpm for 15 min to collect serum. The serum samples were stored at −20°C till further use. Animals were sacrificed on the 116^th^ day and subjected to the following estimation.

### Mammary gland whole mount

The fourth abdominal mammary glands obtained were dissected, stretched onto a slide, placed in a fixative solution and stained with a carmine aluminum solution to prepare whole mounts [20]. Whole mounts were examined under the 4X microscope and evaluated to assess the number of alveolar buds/terminal end buds (AB/TEB). The whole mounts were also evaluated for ductal elongation and differentiation. Ductal elongation was measured, using a ruler, as the distance (in cm) from the nipple to the end of the epithelial tree. Mammary gland differentiation was assessed by scoring the number of alveolar buds (ABs) type 1 and type 2. The score values (0–5) from AB1 and AB2 were added for a final differentiation score (0–10). The average rating values (0–5) from AB1 and AB2 were added to the lobule score values (0–5) for a final differentiation score (0–10) [20].

### Biochemical estimation

The mammary gland tissues (10 % w/v) were homogenized in 0.15 M KCl and centrifuged at 10,000 rpm. The supernatants were scrutinized for biochemical parameters including thiobarbituric acid reactive substances (TBARs), superoxide dismutase (SOD), catalase and glutathione (GSH) using the methods established in our laboratory [21–24].

### Determination of Nitric Oxide (NO) level in serum

Generation of NO in the serum samples was arbitrated by measuring nitrite accumulation, using Griess reagent [1 % sulphanilamide, 0.1 % N-(1-napthyl)-ethylenediamine dihydrochloride in 5 % H_3_P0_4_].

Equal quantity (500 μl) of serum and Griess reagent were mixed and incubated at 37 °C for 5 min. The test mixture was subsequently read on the UV-Visible spectrophotometer (Cary60, Agilent Technologies, CA95051, US) at 540 nm [25].

### Enzymatic activity of COX and LOX

A 10 % mammary gland tissue homogenate in TRIS buffer (50 mM) was centrifuged at 5000 rpm for 5 min followed by sonication. The tissue supernatant (10 μl) was incubated for 5 min with TRIS buffer (160 μl). A 10 μl each of TMPD reagent and arachidonic acid (AA) solution were added and read at 630 nm using a multiplate reader (ALERE Microplate Reader, AM-2100) at 0 and 30 s interval. AA solution was prepared by mixing 50 μl of 40 mM AA with 50 μl of 0.1 N potassium hydroxide using vortexing and subsequently 900 μl of double distilled water. TMPD stock solution was prepared by dissolving 0.3 mg in 1 ml of distilled water and subsequent 1:10 dilution was prepared for the assay [26, 27].

For LOX assay, 25 μl of AA solution was added to the 475 μl supernatant (as prepared for COX assay) and incubated for 6 min. A 500 μl of ferrithiocyanate (FTC) reagent was added and read at 480 nm using U V spectrophotometer (Cary 60, Agilent Technologies International Private Limited, CA United States) after 5 min. FTC reagent was prepared by mixing the reagent 1 (4.5 mM FeSO_4_ in 0.2 M HCl) and reagent 2 (3 % NH_4_SCN methanolic solution) in 1:1 ratio [28].

### Fatty acid methyl ester (FAME) analysis of mammary gland tissue

Mammary gland tissue homogenate (0.5 %) was prepared in the mixture of chloroform: methanol (2:1) by using tissue homogenizer followed by sonication at 4 °C for 5 min. The homogenate was subsequently filtered using a whatmann filter paper and final volume made up by methanol. The filtrate was mixed thoroughly with 0.2 ml volume of double distilled water for removal of non-lipid contaminants. The mixture was kept for 30 min and centrifuged at 5000 rpm for 5 min.; upper phase was removed, and lower phase was collected with mammary gland lipids. Methyl esters for the lipid samples were prepared by stirring 0.5 gm of samples with hexane (2 ml). Methanolic KOH (2 N) (0.2 ml) was added to the above mixture and vortexed for 15 min. The phases were allowed to settle down, and the upper layer containing the FAME was collected [29].

The FAME samples were filtered using 0.2 μm syringe filters and subjected to the gas chromatographic analysis (Perkin Elmer GC- clarus 480; Column : Elite-5 Length-30 mt, Internal diameter- 0.25 mm) using Flame ionization detector(250 °C; carrier gas: nitrogen (10 psi); volume of injection 1 μl; Oven temperature: 150 (1 min), ramp 1–5 °C/min to 230 °C (5 min), ramp 2–150 °C /min to 245 for 12 min; internal standard : cetyl alchohol [30].

### Western blotting

Protein samples were prepared from the mammary gland tissue through acetone precipitation and quantified by using the Bradford reagent [31]. SDS-PAGE analysis was performed following the principles of laemmli with slight modifications [32]. Briefly, protein samples were mixed with sample buffer (125 mM Tris–HCl, pH6.8, 20 % glycerol, 4 % SDS, 0.05 % bromophenol blue, 10 % 2-mecaptoethanol). A 30 μg of protein sample was allowed to resolve through 12 % polyacrylamide gel using SDS-PAGE (GX-SCZ2+, Genetix Biotech Asia Pvt. Ltd, New Delhi). The proteins as resolved through SDS-PAGE were transferred to a PVDF membrane (IPVH 00010 Millipore, Bedford, MA USA) using semidry transfer (GX-ZY3, Genetix Biotech Asia Pvt. Ltd, New Delhi). Subsequently, membrane was blocked with 3 % BSA and 3 % not fat milk in TBST for 2 h and incubated overnight with primary antibody against Pgp 9.5(MA1-83428) (1:2000 dilution), NF-kBP65 (MA5-1616) (1:2000) and β-actin MA5-15739-HRP(1:3000 dilution) (Pierce, Thermo Scientific, USA). The membrane was washed with TBST thrice and incubated with HRP conjugated rat anti-mouse secondary antibody (31430, 1:5000 dilutions) (Pierce Thermo Scientific, USA) at room temperature for 2 h. The signals were detected using an enhanced chemiluminescence substrate (Wester Bright ECL HRP substrate, Advansta, Melanopark, California, US). The Quantification of protein was done through densiometric digital analysis of protein bands using Image J software [32, 33].

## Results

**Fig. 1:**
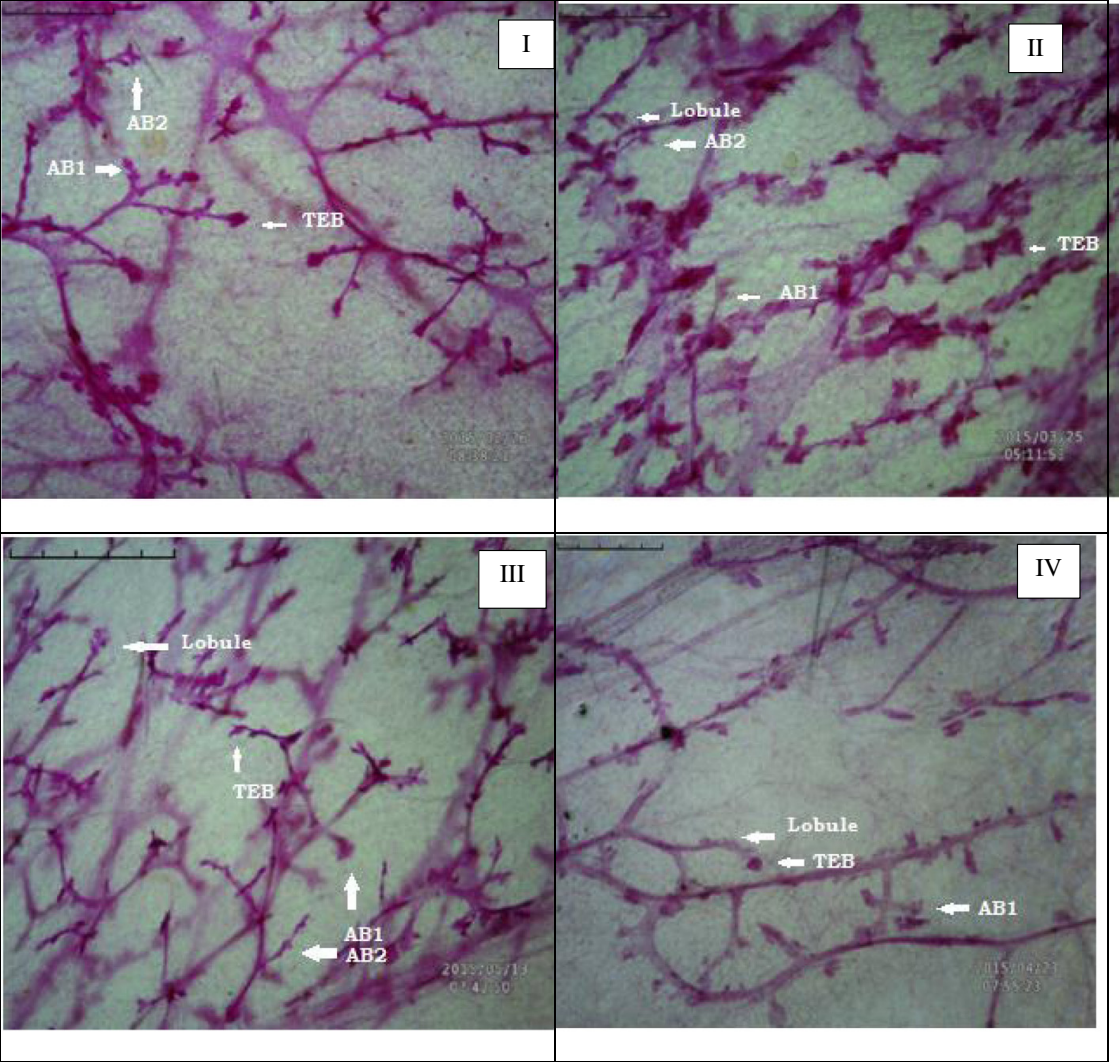
Whole mount of mammary gland tissue subjected to carmine staining: Group I: Control, Group II: MNU, Group III: β-sitosterol (10 mg/kg), Group IV: β-sitosterol (20 mg/kg)

**Fig. 2:**
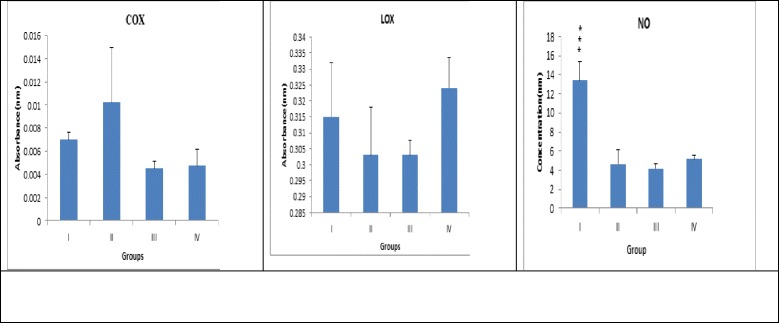
Effect of MNU and β-sitosterol on inflammatory markers and nitric oxide level: GroupI : Control, Group II: MNU, Group III: β-Sitosterol (10 mg/kg), Group IV : β-Sitosterol (20 mg/kg)

**Fig. 3:**
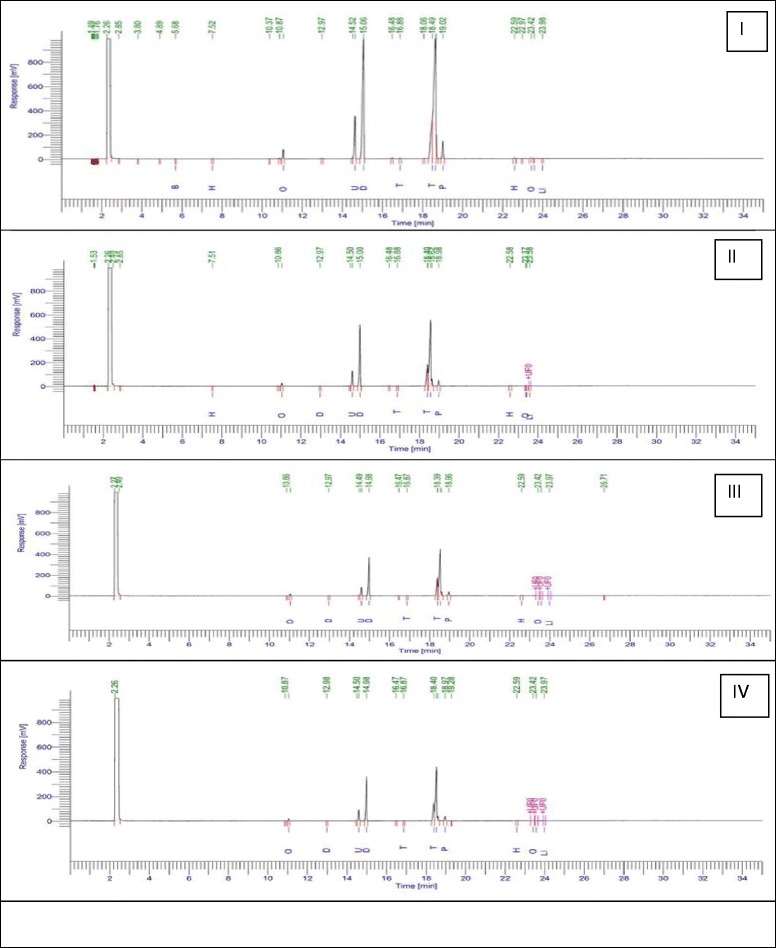
FAME of the mammary gland tissue subjected to MNU and β-sitosterol: Group I: Control, Group II: MNU, Group III: β-sitosterol (10 mg/kg), Group IV: β-sitosterol (20 mg/kg)

**Fig. 4:**
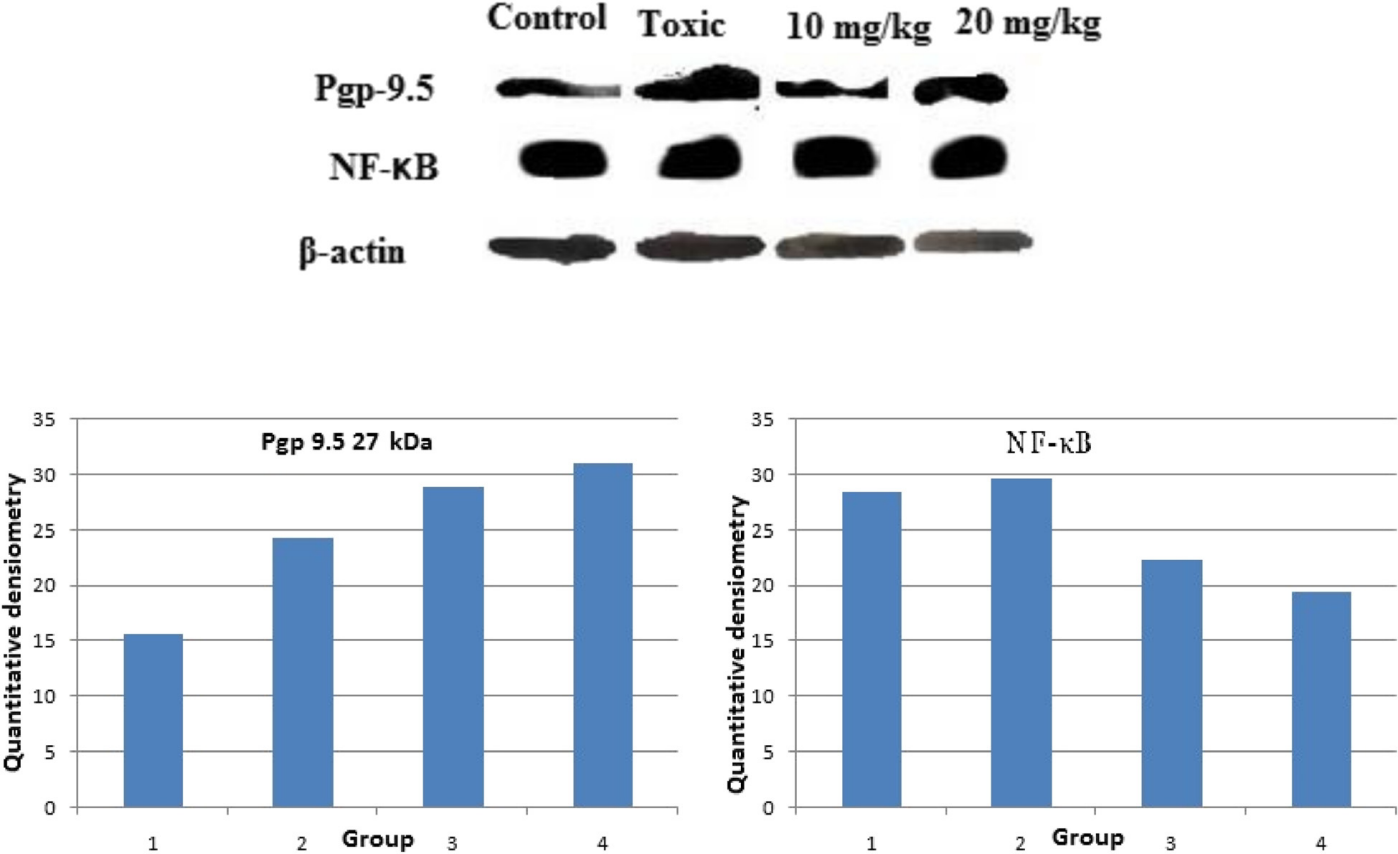
Western Blot analysis: Group I: Control; Group II: MNU; Group III: β-sitosterol10 mg/kg; Group IV: β-sitosterol 20 mg/kg. The western blot analysis containing whole tissue lysate from rat mammary gland was probed for P-gp 9.5 antibody; its immunoreactivity was evident as a band of molecular weight of 27 kDa. In case of control the expression is low in comparison with the toxicant. The low and high dose of β-sitosterol shows a similar pattern of elevated expression in a dose dependent manner. Results of the β-actin analysis are shown as an internal control

The results revealed significant inflation in the AB/TEB’s score with MNU treatment which was dose-dependently curtailed down with the β-sitosterol treatment. A similar pattern of negating effects was evident for the β-sitosterol, when scrutinized for the differentiation score in the whole mount of the mammary gland (Table 1; Fig. 1). The TBARs levels were significantly upraised with MNU administration, and β-sitosterol was evident for a significant curtailment of the same. The tissue catalase, SOD, and GSH levels were diminished after the MNU and high dose of β-sitosterol helped to restore the tissue catalase level without affecting the SOD levels (Table 2). It would be appropriate to remark that low dose of β-sitosterol failed to regulate the oxidative stress markers favorably. In fact low dose of β-sitosterol embarked a diminishing effect on the oxidative stress markers. NO levels were replenished dose dependent way in β-sitosterol treatment but less than sham control, but LOX activity was up-regulated with the higher dose of β-sitosterol (Fig. 2). When contemplated on the accounts of the fatty acid profile of the mammary gland tissues, a significant decrease in the saturated and unsaturated fatty acid was evident with the MNU treatment (Table 3, Fig. 3). The MNU treatment was evident with the increased Pgp levels which were further up-regulated after the β-sitosterol treatment (Fig. 4). Treatment with MNU also afforded up-regulated expression of NF-kB, which was curtailed down after the β-sitosterol treatment (Fig. 4).

**Table 1:** Effect of MNU and β-sitosterol on differentiation of mammary gland

Groups	AB1	AB2	AB1 + AB2	LOBULES	DF SCORE- 1 (AB1 + AB2 + LOBULES)	DF SCORE 2 (LOBULES/AB1 + AB2)
CONTROL (3 ml/kg)	685.75 ± 223.36	367.75 ± 79.88	1053.5 ± 302.45	1805.75 ± 460.39	2859.25 ± 756.67	1.76 ± 0.09
MNU (47 mg/kg)	786.5 ± 20.56	432.5 ± 36.60	1219 ± 57.17	2606 ± 243.72	3825 ± 300.89	2.13 ± 0.09
β-SITOSTEROL + MNU (10 mg/kg + 47 mg/kg)	532 ± 178.53	201.5 ± 48.64*	733.5 ± 227.17	1774.5 ± 417.74	2508 ± 644.92	2.48 ± 0.19
β-SITOSTEROL + MNU (20 mg/kg + 47 mg/kg)	380.5 ± 111.83	143 ± 2.00**	523.5 ± 113.83	1390.5 ± 142.92	1914 ± 256.76	2.72 ± 0.31

(Values are Mean ± SEM), each group contains eight animals. Comparisons were made on the basis of the one-way Anova followed by Bonferroni test. All groups were compared to the toxic control group (*p < 0.05, **p < 0.01)

**Table 2:** Effect of β-sitosterol upon various in-vivo antioxidant markers

Groups	TBARs (nM of MDA/μg of protein)	GSH (mg %)	SOD (Units of SOD/mg of protein)	Catalase (nM of H2O2/min/mg of protein)
CONTROL (3 ml/kg)	86.08 ± 5.00***	2.30 ± 0.12	1.92 ± 0	50.63 ± 7.81*
MNU (47 mg/kg)	118.84 ± 4.65	2.00 ± 0.16	1.81 ± 0.02	31.76 ± 2.59
β-SITOSTEROL + MNU (10 mg/kg + 47 mg/kg)	147.93 ± 6.67**	1.03 ± 0.14**	1.82 ± 0.03	26.90 ± 3.06
β-SITOSTEROL + MNU (20 mg/kg + 47 mg/kg)	105.51 ± 2.35	1.24 ± 0.21*	1.80 ± 0.03	51.75 ± 3.31*

(Values are Mean ± SEM), each group contains eight animals. Comparisons were made on the basis of the one-way ANOVA followed by Bonferroni test. All groups were compared to the toxic control group (*p < 0.05, **p < 0.01, ***p < 0.001)

**Table 3:** Fatty acid profiling of mammary gland tissue treated with MNU and β-Sitosterol

S no.	Type of fatty acid			Control	MNU (47 mg/kg)	β-sitosterol (10 mg/kg)	β-sitosterol (20 mg/kg)
	Name	Saturated	Unsaturated				
1	C4:00	Butanoic acid		0.01	-	-	-
2	C6:00	Hexanoic acid		0.04	0.02	-	-
3	C8:00	Octanoic acid		0.80	0.44	0.36	0.31
4	C10:0	Decanoic acid		-	0.05	0.05	0.03
5	C11:0	Undecanoic acid		4.65	2.34	1.64	1.62
6	C12:0	Dodecanoic acid		18.08	10.47	7.94	6.92
7	C13:0	Tridecanoic acid		0.07	0.05	0.04	0.03
8	C14:0	Tetradecanoic acid		6.52	3.36	2.30	3.90
9	C14:1		Myristoleic acid(ω-3)	27.68	16.14	13.35	10.83
10	C15:0	Pentadecanoic acid		1.98	1.06	0.96	0.18
11	C17:1	Heptadecanoic acid		0.13	0.10	0.14	0.06
12	C18:0	Octadecanoic acid		0.01	0.00	0.07	0.08
13	C18:1		Oleic acid(ω-9)	0.00	0.03	0.01	0.03
14	C18:2		Linoleic acid (ω-6)	0.02	0.03	0.02	0.02
Total fatty acid				59.94	34.09	26.88	24.64
Saturated fatty acid				32.24	17.89	13.5	13.76
Unsaturated fatty acid				27.7	16.2	13.38	10.88

## Discussion

The present study perceived critical protection by β-sitosterol to combat the deleterious effects of MNU on the mammary gland. The whole mount preparations are frequently used as a convenient method for the examination of small proliferative lesions as represented by an increase in the number of terminal end buds (TEBs) [34]. The AB/TEB is the morphologic structure reported to be the target of carcinogenic outcome in the rat mammary gland carcinoma and also the structure from which premalignant pathologies changes in ductal carcinoma [35]. We considered it primary to determine whether the vascularisation and angiogenesis of TEBs was affected by MNU administration. If angiogenesis was increased, then a decrease in vascularity of TEB by a cancer preventive agent is expected. Therefore, TEBs/AB is potentially used as an early positive marker of antiangiogenic cancer preventive activity [36]. The MNU treatment was evident with a rise in AB/TEBs count and differentiation score, and can be closely linked to increased risk of mammary tumourigenesis. These identical mammary structures are the sites of malignant transformation in the rat mammary gland [34] and the corresponding structures in the human breast are the terminal ductal lobular units [37]. Studies have shown that higher number of AB/TEBs correlates with greater risk of mammary cancer [34]. Treatment with MNU was evident with an increase in some alveolar bud score and differentiation score which was counteracted by β-sitosterol to the sizable amount in a dose-dependent manner.

Chronic inflammation and release of NO results in DNA damage and nitrosylation of proteins which has been implicated in carcinogenesis. The COX and LOX inhibitors have been deliberated to have anticancer activity since long [38]. Infact, over expression of COX and LOX in progression and neo-angiogenesis of human cancer has been supported by large body of scientific literature [39, 40]. A recent finding also affirmed that the COX-2 over expression can induce mammary gland carcinogenesis by reducing the proapoptotic (BAX and BCL-XL) and inflating the antiapoptotic proteins (BCL-2) expression in the mammary gland tissue [41, 42]. Overexpression of LOX has also been reported in variety of tumors including breast, colorectal and prostate cancer [43, 44]. In line with the previous reports upregulated COX levels were perceived after MNU treatment and β-sitosterol afforded a marked reduction of the same. However, MNU treatment downregulated LOXs in the course of tumor development suggesting that LOX exhibit antitumorigenic rather protumorigenic effects. Infact decreased LOX activity has been reported in the prostate cancer, high-grade prostatic intraepithelial neoplasia and human colon cancer and therefore represent a research question which needs to be addressed to its full [45–47].

NF-kB is a group of sequence-specific transcription factor and is best known as a key regulator of inflammatory responses [48]. Elevated NF-kB DNA binding activity is detected in both mammary carcinoma cell lines and primary human breast cancer tissues. In fact, NF-kB is reported to contribute expansion of breast tumor stem cells and enhancement of vasculogenesis [49] and, therefore, has emerged as a viable target for cancer progression. In the same line, we perceived significant up-regulation of the levels of NF-kB in MNU treated animals, which subsided to a significant amount by β-sitosterol treatment. The efficacy of β-sitosterol to nudge down NF-kB is in agreement with the previous findings [49]. Prima faci it appears that β-sitosterol can impart a noticeable effect against the MNU induced carcinogenesis.

The link between MNU induced carcinogenesis and oxidative stress regulators is well established [50, 51]. The oxidative stress is defined as an imbalance in the production of reactive oxygen species (ROS) and reactive nitrogen species (RNS) leading to impaired cellular metabolism and changes in the intra and extracellular environmental conditions. ROS and RNS can further lead to DNA damage such as mutations, deletions, amplifications and rearrangements which can lead to cancer initiation and progression. The effects of ROS and RNS are balanced by the exogenous/endogenous antioxidants acting through enzymatic or non-enzymatic mechanisms. Treatment with MNU deliberated significant rise in TBARs with noticeable downturn in the enzymatic defense of SOD/catalase/GSH. The NO levels were also curtailed down subsequent to MNU treatment. The drop in the oxidative defense and up-regulation of the lipid peroxidation products is a clear testimony of oxidative stress and is in line with the previous reports about MNU induced carcinogenesis [52, 53]. Treatment with β-sitosterol demarcated significant restoration of the oxidative stress markers at a higher dose. Interestingly, β-sitosterol at lower dose deteriorated the markers of oxidative stress, in particular, TBARs, GSH and enzymatic levels of catalase. Authors would not like to mention that the results scrutinized with the low dose of β-sitosterol are neither in line with the previous reports, nor in consonance with the findings elaborated by us in the preceding section of the manuscript. All in all, one can conclude that β-sitosterol can impart a demarcating biochemical effect on the MNU induced carcinogenesis through modifying the inflammatory signaling and regulating the oxidative stress markers at higher dose.

Pgp-9.5 (ubiquitin COOH-terminal esterase L1 or UCHL-1) is an ubiquitin COOH terminal hydrolase that is widely expressed in different type of cancer cells [18]. Recent study demonstrated that Pgp-9.5 is highly overexpressed at many non-neuronal tumors, including breast, colorectal and pancreatic tumors [19]. There are very few studies, carried out in the particular subtype of ER positive breast cancers and result are also non-conclusive [54]. PGP-9.5 expression is significantly associated with higher MVD (intratumoral microvessel density) in ER negative breast cancer [55]. Their actual role and underlying mechanism is not properly understood in case of ER or PR receptor positive breast cancer produced by MNU treatment [54]. In this particular study the toxic group showed elevated expression in comparison with control, which ensures overexpression of Pgp-9.5 in MNU induced toxicant group. Pgp-9.5 overexpression in the primary cancerous tissues is due to biological transformations, imparting it the status of oncogenic marker [56–58]. UCHL-1 encodes two opposite characteristic pattern in the ubiquitin pathway. When ubiquitin COOH terminus hydrolase to generate another single ubiquitin, the other end show ligase activity which lead to multiple ubiquitination. Different animal studied showed that on application of apoptotic stimuli, UCHL-1 overexpression does not happen [59]. The previous results suggest that Pgp-9.5 is more likely to be act as a tumor suppressor molecule, which is in line with our experimental outcome [60–62]. The dose dependent increase in Pgp-9.5 expression was observed after β-sitosterol treatment.

Fatty acids are important modifiers of the mammary gland carcinoma risk and are composed of complex mixtures including the saturated, monounsaturated and polyunsaturated fatty acids. Modifying the fatty acid profile has been found to be closely associated with mammary gland cancer risk in rodent models and humans [63–65]. Plethoras of scientific studies are available pointing the positive association between saturated/monounsaturated fatty acid and progression of mammary gland carcinoma, with a negative modulation by polyunsaturated fatty acid. Similar framework of decreased total polyunsaturated fats and increased saturated/monounsaturated fat in the mammary gland was scrutinized after the MNU treatment. β-sitosterol afforded to curtail down the upraised saturated/monounsaturated fats profile in the mammary gland tissue dose-dependently without much affecting the total PUFA concentration. It would be appropriate to mention that total fat in a cancerous tissue has been directly correlated drug resistance and subsequently Pgp expression. In fact inhibition of the multidrug resistance, MDR1/P-gp has been postulated as one of the mechanism through which polyunsaturated fatty acids exert a synergetic effect in the response of tumor cells to anticancer drugs [66]. In the present study, we contemplated overexpressed Pgp and diminished unsaturated fatty acid after the MNU treatment and vice versa results after the β-sitosterol administration. The up-regulated PUFA levels after the β-sitosterol treatment also strengthens the previous observations that β-sitosterol can promote apoptosis through activating FAS enzyme.

## Conclusion

With all above one can conclude that β-sitosterol can impart significant protection against the MNU induced mammary gland carcinogenesis through modifying the pathological, biochemical and inflammatory markers, which authors would like to attribute to its modulatory activity towards Pgp and fatty acid profile of the mammary gland. Authors would also like to hypothesize that the same could be attributed to its potential to activate FAS and thereby promote apoptosis. However, because FAS is primarily regulated through the hypoxia-inducible factor and subsequently prolyl hydroxylase, it would interest to investigate the effect of β-sitosterol on the same.

## Abbreviations

AA, arachidonic acid; AB/TEB, alveolar buds/terminal end buds; CAT, catalase; COX, cyclooxygenase; FAME, fatty acid methyl ester; FAS, fatty acid synthase; FTC, ferrithiocynate; GSH, glutathione; LOX, lipoxygenase; MNU, methyl nitrosourea; MVD, intratumoral microvessel density; NO, nitric oxide; RNS, reactive nitrogen species; ROS, reactive oxygen species; SOD, superoxide dismutase; TBARs, thiobarbituric acid reactive substances; TMPD, N, N, N’, N’-tetramethyl-p-phenylenediamine
